# Experiences of People with Multiple Sclerosis in Sensor-Based Jump Assessment

**DOI:** 10.3390/bioengineering12060610

**Published:** 2025-06-03

**Authors:** Anne Geßner, Anikó Vágó, Heidi Stölzer-Hutsch, Dirk Schriefer, Maximilian Hartmann, Katrin Trentzsch, Tjalf Ziemssen

**Affiliations:** Center of Clinical Neuroscience, Department of Neurology, Faculty of Medicine and University Hospital Carl Gustav Carus, TUD Dresden University of Technology, Fetscherstraße 74, 01307 Dresden, Germany; anne.gessner@uniklinikum-dresden.de (A.G.); ani.v@web.de (A.V.); dirk.schriefer@uniklinikum-dresden.de (D.S.);

**Keywords:** jump assessment, sensor-based, biomechanical analysis, patient experience, multiple sclerosis

## Abstract

(1) Background: When implementing new biomechanical and technology-based assessments, such as the jump assessment in Multiple Sclerosis (MS), into clinical routine, it is important to ensure that they are based on the real needs of patients and to identify and adapt to potential barriers early on. (2) Methods: In the present cross-sectional study, 157 pwMS performed a sensor-based jump assessment on a force plate consisting of three jump tests: 10 s jump test (10SHT), countermovement jumps (CMJ), and single-leg countermovement jumps (SLCMJ). After the jump assessment, the patient experience measures (PREM) were recorded using a paper-based questionnaire on an 11-point scale from 0 (positive) to 10 (negative). (3) Results: PwMS showed an overall positive experience with the sensor-based jump assessment. “Staff support performance”, “acceptance required time”, “usefulness” of the results, and “integration of results in therapy” were the best rated items with a median of 0 (positive). The CMJ was perceived as the easy (*p* < 0.05) and less exhausting (*p* < 0.05). PwMS who experienced CMJ as easy, not exhausting, and safe were associated with higher CMJ performance, especially in peak power, flight time, and jump height (r > −0.4). Significant associations were found between PREMs and age, sex, BMI, physical activity, and disability degree. (4) Conclusions: The study findings support the feasibility of jump assessment in clinical practice and highlight the need for patient-centered integration of innovative technologies to optimize precision neuromuscular function evaluation in MS.

## 1. Introduction

Multiple sclerosis (MS) is a neurodegenerative disease characterized by inflammatory-mediated processes throughout the central nervous system resulting in a heterogeneous symptomatic presentation and clinical progression with motor, sensory, and cognitive impairments [1]. One of these motor impairments is a reduction in neuromuscular and muscle mechanical function (MMF) (i.e., muscle strength and muscle power) [2,3]. Especially during fast dynamic muscle contractions, people with Multiple Sclerosis (pwMS) show a decrease in lower limb muscle strength, power, and rate of force compared to healthy controls (HC) [3,4]. These impairments in neuromuscular function have critical implications across all components of the International Classification of Functioning, Disability, and Health model, particularly impacting the activity level in pwMS [5]. Several studies have shown that reduced muscle function is associated with impaired functional ability, activities of daily living, and quality of life [2,3,6]. The integration of jumping assessments as a digital biomarker in our MS digital twin framework offers a promising approach for enhancing the management of MS, particularly in patients with low disability levels [7]. This approach supports proactive disease monitoring and personalized treatment interventions [8].

The precise collection of data reflecting neuromuscular function in pwMS enables a meaningful assessment in addition to standard clinical outcome measures (i.e., Expanded Disability Status Scale (EDSS)) and creates a detailed motor function patient profile [9,10,11]. Among the many methods (i.e., isokinetic dynamometry, MMF-tests) being used to assess lower limb neuromuscular function, the sensor-based jump assessment on a force plate represents a novel, high-challenge, and objective assessment tool in MS, aligning with recent advancements in biomedical technology for precise motor function evaluation. It enables the identification of subtle neuromuscular impairments in MS that fall below the clinical threshold of the EDSS [4,12,13]. Based on these findings, jump assessment on a force plate offers a comprehensive and objective approach to measuring motor function, and it is a sensitive method for assessing lower extremity neuromuscular function and asymmetries in MS [4,13,14,15].

While the potential of the jump assessment as a novel assessment tool in MS is promising, the integration of innovative technologies into clinical practice requires a patient-centered approach. Understanding patients’ experiences of such novel assessments is essential to ensure their usability and long-term implementation into clinical routines, contributing to personalized treatment and rehabilitation strategies. However, the patient’s experience of its use in MS has not been adequately addressed in the existing literature. A combined collection of functional assessments (i.e., jump and gait analysis), standard clinical outcome measures (i.e., EDSS and Multiple Sclerosis Functional Composite), and patients’ experiences may serve as an indicator of data quality, as satisfied patients are more likely to be active participants in the treatment and management of their disease, adhere to treatment and have a higher quality of life [16]. Thus, it is important to understand patients’ perspectives when trying to improve the quality of treatment and healthcare services for patient-centered care in MS [17]. It is also essential to capture patient experience when implementing new biomechanical and technology-driven assessments in the clinical routine, such as the jump assessment, to ensure that they are based on the real needs of patients and to identify and adapt potential barriers (e.g., time and uncertainty) at an early stage. This is provided by “Patient-reported experience measures” (PREMs)—a type of survey measurement tool used in health research that aims to capture patients’ experiences and perspectives of care [18]. Several studies have been used in research to evaluate the experience of pwMS to determine patients’ experience of diagnosis and management, communication with healthcare professionals and healthcare services [19,20,21].

We aimed in this exploratory study first (I) to evaluate the patient-reported experience of sensor-based jump assessment in pwMS, and secondly (II) to investigate the association of various influencing factors (e.g., clinical, demographic and arthrometric parameters) on the patient-reported experience of sensor-based jump assessment to identify barriers and facilitators of ensure the usability and long-term implementation into clinical routines.

## 2. Materials and Methods

### 2.1. Participants

We conducted a cross-sectional study at the MS Center of the Center of Clinical Neuroscience of the Department of Neurology, University Hospital Carl Gustav Carus, Dresden, Germany. Between June and December 2023, we investigated 157 pwMS admitted to our department. Inclusion criteria were (1) a confirmed diagnosis of multiple sclerosis according to McDonald criteria, (2) an EDSS Score between 0 and 5.5, (3) an age between 18 and 50 years, (4) written informed consent (5) the ability to walk > 500 m without an aid, and (6) the ability to perform heel stand, toe stand, and squats. Exclusion criteria were the presence of any orthopedic or surgical condition affecting jumping ability. Patients with the fear of insecurity during jumping and pregnancy were also excluded. The study was approved by the local ethics committee (BO-EK-320062021) and all participants provided written informed consent for their participation.

### 2.2. Measures and Procedures

#### 2.2.1. Sensor-Based Jump Assessment

**Figure 1:**
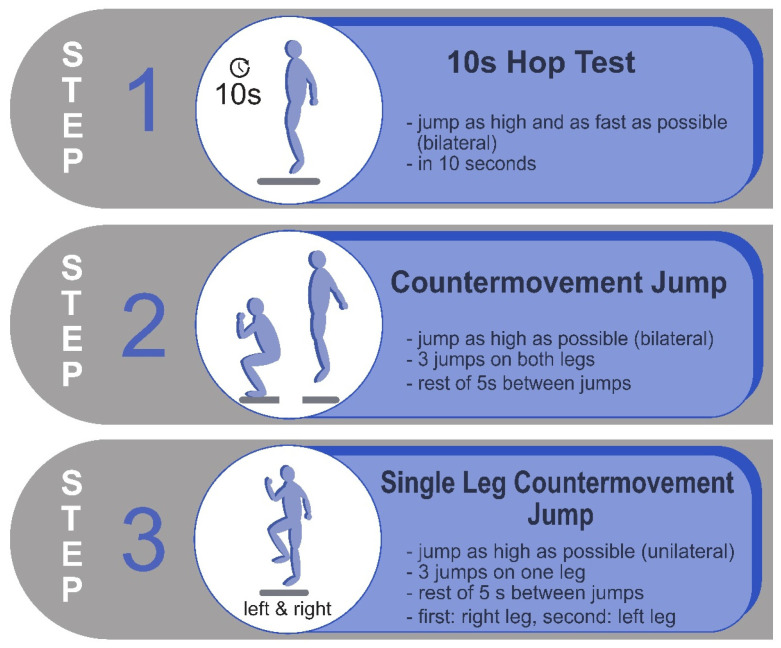
Description of the sensor-based jump assessment on a force plate for pwMS. Prior to the jumping tests, a physiotherapist explained and demonstrated the jumping technique for each jump test to each participant. Prior to data collection, all participants practiced a test jump. All jump tests were performed without arm swing and using everyday clothing and socks. Between the three jump tests was a rest of 2–3 min.

The MS Center at the Center of Clinical Neuroscience employs a three-pronged approach to assess jumping abilities. The sensor-based jump assessment comprises three tests: the 10 s hop test (10SHT), the countermovement jump (CMJ) and the single-leg countermovement jump (SLCMJ) (right and left separately) (see Figure 1). All tests were performed on a portable single force plate (1016 length × 762 width × 127 mm height) by AMTI (Advanced Mechanical Technology Inc., Watertown, MA, USA, AccuPower-O), while three-dimensional ground reaction forces (Fx, Fy, Fz) and force moments (Mx, My, Mz) were recorded at a frequency of 1000 Hz. The biomechanical analysis software AccuPower Solutions (Version 1.5.4.2082) was used to analyze the jump parameters from the recorded ground reaction forces of the force plate. The jumping parameters were selected based on insights derived from prior studies in MS (4,15) and sports science (31). The sensor-based jump assessment was conducted by three raters.

#### 2.2.2. Patient-Reported Experience Measures (PREM) Questionnaire

Following the completion of the jump assessment, pwMS were asked by staff whether they agreed to participate in a PREM questionnaire to provide a comprehensive insight into various dimensions of their experience and to evaluate the functionality, user orientation, and practicability of the jump assessment. Participants completed the PREM questionnaire self-administered to mitigate the potential influence of examiner bias.

The paper-based PREM questionnaire included ten questions on whether conducting the sensor-based jump assessment (total jump assessment and individual jump tests) was easy, questions on whether conducting the jump assessment (total jump assessment and individual jump tests) was straining, two questions about the comfort, three questions to rate the usefulness and relevance, one question to evaluate the staff support, and two questions assessing the appropriateness of time and frequency of the jump assessment (see Table 1). An 11-point scale from 0 (positive) to 10 (negative) was used for each question. The staff explained the meaning of the 11-point scale in detail to the participants, including the interpretation of the endpoints and intermediate values. Table 1 shows the questions and the interpretation of the rating scale. In addition, the PREM questionnaire asked pwMS in a yes/no format whether they had received physiotherapy for lower limb motor deficits, their current fear of falling while walking, and whether they had fallen in the previous three weeks.

#### 2.2.3. Godin Leisure Time Exercise Questionnaire (GLTEQ)

The GLTEQ is a validated patient-reported outcome measure (PRO) for assessing simple and effective physical activity in pwMS [22,23]. It is a three-item questionnaire to record the frequency at which the subjects performed physically strenuous, moderate, and mild exercise per week in their leisure time. A GLTEQ score of less than 14 units indicates insufficient activity, 14 to 23 indicates moderate activity, and 24 units or more indicates high activity [24].

#### 2.2.4. Expanded Disability Status Scale

To examine the clinical status, the EDSS including pyramidal functional system score (FSS), cerebellar FSS, sensory FSS and ambulation score was assessed using neurostatus by certified raters [25]. The EDSS is the most used disability scale in MS and is well established among neurologists [26].

### 2.3. Statistical Analysis

The distribution of all parameters was visually checked via histograms and Q-Q plots and the assessment of normality was supplemented with the Shapiro–Wilk test. Continuous data are presented as mean ± standard deviations (SD) or medians ± interquartile range (IQR), as applicable. Absolute numbers and percentages were used to describe categorical variables. PREM data were reported descriptively and expressed as median (IQR) or frequency of participants in percent (%). Non-parametric paired Wilcoxon tests were used to assess the differences between the jump tests according to patient experience of strain and difficulty for all pwMS. For additional analysis, the pwMS were divided into subgroups according to physiotherapy (pwMS with and without physiotherapy), fear of falling (pwMS with and without fear of falling), and falls (pwMS with and without falls in the previous three weeks). To evaluate the differences between these pwMS subgroups on the PREM items the non-parametric, Mann–Whitney-U-Tests were used. We applied a Bonferroni–Holm correction to counteract the problem of multiple comparisons. The relationship between each of the PREM items and the continuous variables clinical, demographic and arthrometric parameters, physical activity and CMJ parameters were examined by Spearman’s rank correlation coefficients. Correlations coefficients of |*p*| = 0.10 are considered as low correlation, values of |*p*| = 0.30 as moderate correlation, and values of |*p*| = 0.50 as high correlation [27]. The level of significance was set at *p* < 0.05. Statistical analyses were performed using IBM Statistical Package for the Social Sciences (SPSS) for Windows, version 28 (IBM Corp, Armonk, NY, USA).

## 3. Results

### 3.1. Participants

In total, 157 pwMS agreed to participate in the PREM questionnaire after performing the sensor-based jump assessment. Nine pwMS (5.7%) did not complete the PREM questionnaire in full. All pwMS completed the 10SHT and CMJ during the jump assessment. Six pwMS (3.8%) were unable to perform the SLCMJ due to risk of falling and muscle weakness. Disability status (EDSS) ranged from 0 to 5.5. Participants were mostly female (73.2%) and physical active (72.1%). In all, 26.7% of the participants received physiotherapy for lower limb motor deficits, 14% were concerned about falling, and 5.7% had fallen in the previous three weeks. Participant characteristics are shown in Table 2.

#### PREM Item Outcomes for Sensor-Based Jump Assessment

**Figure 2:**
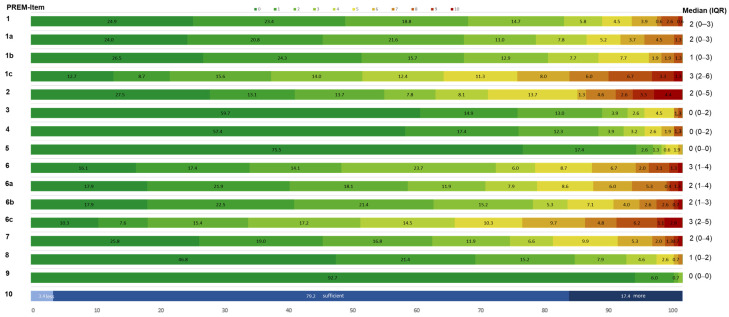
Patient experience in percentage (%) of the PREM items of the jump assessment in pwMS. The colors represent the response scale from 0 (green) to 10 (red) of the patient survey PREM questionnaire. Abbreviations: 1 = difficulty total, 1a = difficulty 10 SHT, 1b = difficulty CMJ, 1c = difficulty SLCMJ, 2 = self-use results, 3 = usefulness results, 4 = integration results in therapy, 5 = acceptance required time, 6 = strain total, 6a = strain 10SHT, 6b = strain CMJ, 6c = strain SLCMJ, 7 = safety, 8 = comfort, 9 = staff support performance, 10 = rating of implementation frequency (description of the PREM items see Table 1), 10SHT = 10-Second-Hop-Test, CMJ = countermovement jump, SLCMJ = single-leg countermovement jump.

**Figure 3:**
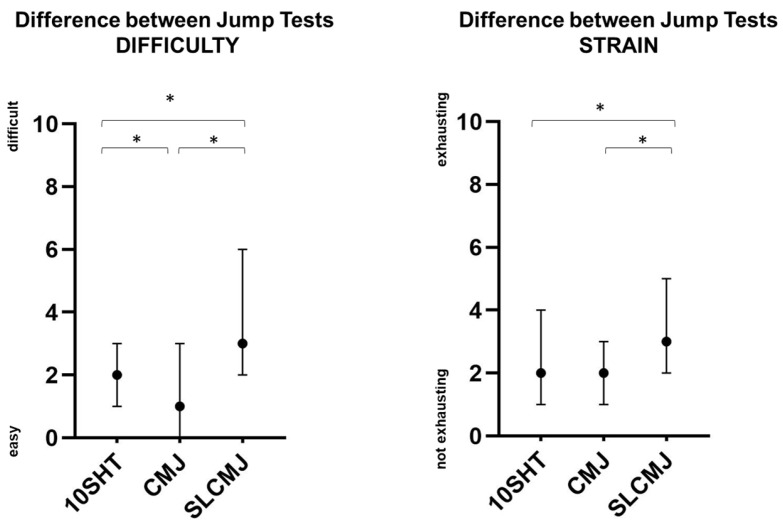
Box-Plots (median, IQR) of perceived difficulty and strain for the jump tests in pwMS, * = significant difference between the jump tests (*p* < 0.05) according to Wilcoxon Test. Abbreviations: 10SHT = 10-Second-Hop-Test, CMJ = countermovement jump, SLCMJ = single-leg countermovement jump.

The median score for each PREM item was between 0 and 3.0 (out of 10), indicating an overall positive patient experience of the sensor-based jump assessment (see Figure 2. The PREM items “staff support performance”, “acceptance required time”, “usefulness of the results”, and “integration of results in therapy” were the best rated items with a median of 0 (very good, appropriate, convinced, and useful). PwMS tended to report the jump assessment as being comfortable (median 1; IQR: 0–2) and safe (median: 2, IQR: 0–4). Most patients experienced the total jump assessment as easy (median 2; IQR: 0–3) and not very exhausting (median 3; IQR: 1–4). The SLCMJ was experienced as the significantly most difficult (SLCMJ vs. 10SHT: Z = −7.735, *p* < 0.001; SLCMJ vs. CMJ: Z = −8.989, *p* < 0.001) and exhausting (SLCMJ vs. 10SHT: Z = −6.600, *p* < 0.001; SLCMJ vs. CMJ: Z = −8.797, *p* < 0.001) jump test, followed by the 10SHT and the CMJ (see Figure 3). An annual implementation was considered sufficient by 79.2% of pwMS. Figure 2 shows the percentage distribution of PREMS response frequencies.

### 3.2. Association Between PREMs and Physiotherapy Received, Fear of Falling, and Falls in the Previous Three Weeks

**Figure 4:**
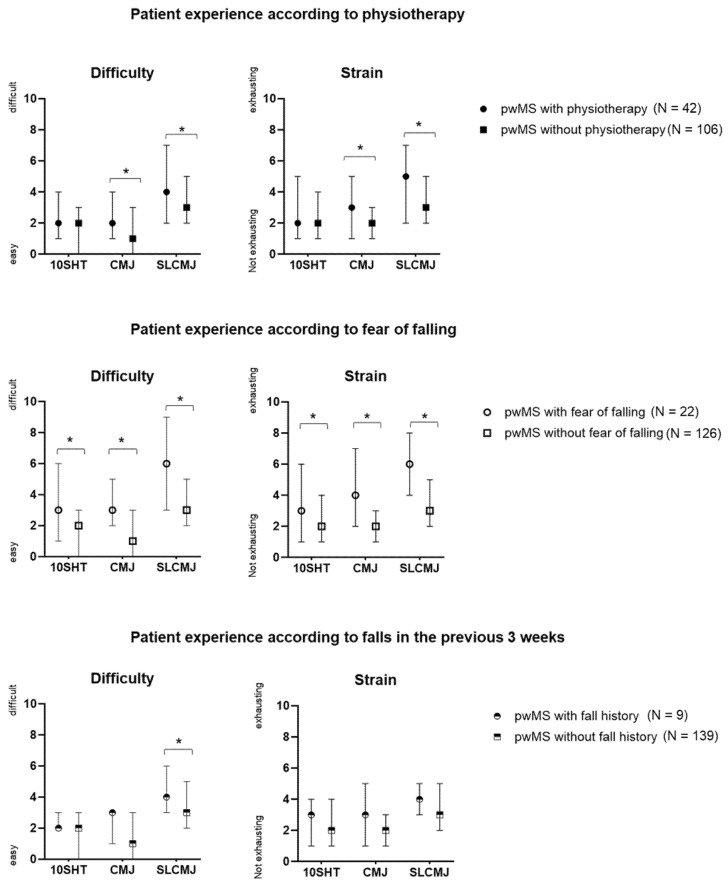
Patient experience of perceived difficulty and strain (median, IQR) for the three jump tests according to physiotherapy, fear of falling, and falls in the previous three weeks. * = significant difference (*p* < 0.05) according to Mann–Whitney-U-Test. Abbreviations: 10SHT = 10-Second-Hop-Test, CMJ = countermovement jump, SLCMJ = single-leg countermovement jump.

PwMS receiving physiotherapy for lower limb motor deficits (N = 42) were significantly more convinced that the results of the jump assessment were useful for their progress monitoring (Z = −2.602, *p* = 0.009), integrated into their therapy (Z = −2651, *p* = 0.008), felt significantly safer (Z = −4.393, *p* < 0.001), and rated the comfort of the jump assessment higher (Z = −2.487, *p* = 0.013) than pwMS without physiotherapy (N = 106). PwMS with physiotherapy found the CMJ and SLCMJ significantly more difficult (CMJ: Z = −3.126, *p* = 0.002; SLCMJ: Z = −2.261, *p* = 0.024) and exhausting (CMJ: Z = −3.945, *p* < 0.001; SLCMJ: Z = −3.684, *p* < 0.001) compared to pwMS not receiving physiotherapy (Figure 4). PwMS with fear of falling (N = 22) perceived the individual jump tests as significantly more difficult (10SHT: Z = −3.631, *p* < 0.001; CMJ: Z = −5.967, *p* < 0.001, SLCMJ: Z = −4.902, *p* < 0.001) and exhausting (10SHT: Z = −3.023, *p* = 0.003; CMJ: Z = −5.783, *p* < 0.001, SLCMJ: Z = −5.447, *p* < 0.001) than pwMS without fear of falling (N = 126) (Figure 4). In addition, pwMS with fear of falling showed significantly more uncertainty (Z = −6.470, *p* < 0.001), less comfort in performing the jump assessment (Z = −3.199, *p* = 0.001), and rated the support and explanation by the study staff as bad (Z = −2.599, *p* = 0.009). PwMS who had fallen in the previous three weeks (N = 9) used their results significantly more frequently for self-monitoring (Z = −2.558, *p* = 0.009) and found significantly (Z = −4.110, *p* < 0.001) that the jump assessment should be more frequent than one a year compared to pwMS who had not fallen in the previous three weeks (N = 139). PwMS with falls in the previous three weeks found the SLCMJ significantly more difficult than pwMS with no falls in the previous three weeks (Z = −2.605, *p* = 0.009) (Figure 4). Figure 4 shows the patient experience of difficulty and strain for the three jump tests according to physiotherapy, fear of falling, and falls in the previous three weeks. Appendix A Table A1 shows the median (IQR) for pwMS regarding to physiotherapy, fear of falling, and falls in the previous three weeks.

#### 3.2.1. Association Between PREMs and Clinical Outcomes

Overall, the bivariate correlation between PREM items and clinical showed mild to moderate associations (Table 3). The highest correlation coefficients between PREM items and clinical outcomes were found between the strain of the CMJ and the FFS pyramidal (r = 0.362), FFS cerebellar (r = 0.334), and ambulation score (r = 0.386) of the EDSS. A higher EDSS score correlated with more difficulty, strain, and insecurity in the total jump assessment. There were no significant correlations between the PREM items “self-use of results”, “usefulness of results”, “integration of results in therapy”, and “acceptance required time” with the EDSS score. No significant correlation was found between disease duration and PREM items, except that longer disease duration was associated with significantly more self-use of jump assessment results (r = 0.141). Correlations for PREM items and clinical outcomes are reported in Table 3. The median (IQR) of the PREM items for EDSS subgroups are shown in Appendix A Table A2.

#### 3.2.2. Association Between PREMs and Age, BMI, Sex, and Physical Activity

The bivariate correlation between PREM items and age, BMI, sex, and physical activity showed mild associations (Table 3). Higher age was significantly associated with higher difficulties (r = 0.093) and strain (r = 0.118) in the jump assessment as well as less usefulness of the results (r = −0.144) (see Table 3). A similar correlation was found between BMI and the PREM items, where higher BMI correlates significantly with higher difficulty (r = 0.110) and strain (r = 0.237), as well as with higher uncertainty (r = 0.169) and less comfort (r = 0.095) of jump assessment. Small significant correlations were found between sex and the PREM items. Males found the jump assessment significantly easier and less strenuous and rated the safety and comfort higher than females (see Appendix A Table A2). PwMS with higher physical activity were significantly correlated with lower strain in the 10SHT, CMJ, and SLCMJ. Correlations between PREMs and age, sex, BMI, and physical activity are summarized in Table 3. The median (IQR) of the PREM items for age, sex, and physical activity subgroups are shown in Appendix A Table A2.

### 3.3. Association Between PREM Items and CMJ Parameters

**Figure 5:**
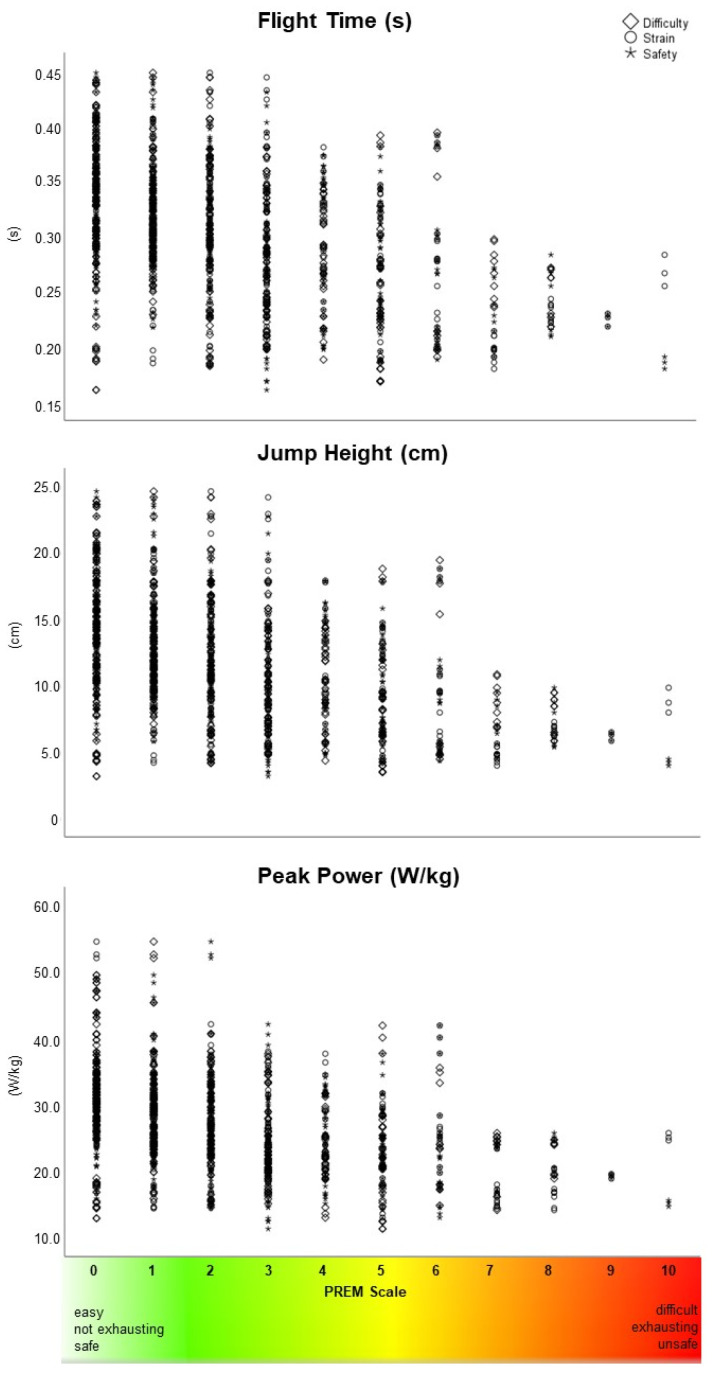
Scatterplots of correlation between the PREM items difficulty, strain, and safety with the CMJ jump parameters (continuous) flight time, jump height, and peak power.

Overall, the bivariate correlation between PREM items and CMJ parameters showed mild to moderate associations (Table 4). The highest correlation coefficients (r > −0.4) were found between difficulty, strain of the CMJ, and the safety of the jump assessment with the CMJ parameter peak power, flight time, and jump height (Figure 5). PwMS with lower peak force, peak power, and jump height showed significantly more self-use and usefulness of the results for therapy. In addition, pwMS with higher jump performance rated the staff support performance significantly better.

## 4. Discussion

In this cross-sectional study, we investigated the patient-reported experience of sensor-based jump assessment in pwMS. To our knowledge, this is the first study to investigate patients’ experiences of participating in a sensor-based jump assessment in MS.

Overall, our study results demonstrated that pwMS had a positive experience with the sensor-based jump assessment in a clinical setting. “Staff support performance”, “acceptance required time”, “usefulness” of the results, and “integration of results in therapy” were the best rated items. In addition, the positive experiences of the pwMS regarding the comfort and safety of the jump assessment indicate that the jump assessment is well accepted and does not represent a burden for pwMS. This is a crucial factor for successful implementation in clinical practice, as it increases the likelihood of patients undergoing the assessment regularly and integrating them into the monitoring process. This is further supported by the result that most pwMS (79.2%) would like to perform the jump assessment once a year. Although the current annual frequency seems sufficient, additional jump assessment before and after exercise therapy or the integration of continuous measurements in daily life could improve MS monitoring. Such an approach might allow for closer monitoring of pwMS, potentially leading to a more detailed analysis of disease progression and earlier detection of changes in neuromuscular and biomechanical function.

A standardized test procedure, like the presented jump assessment, is essential to make the results comparable in the monitoring in pwMS [28]. On the other hand, an adaptive design of the test protocol according to the patient’s performance is recommended. The jump assessment at CCN Dresden comprise a variety of jump tests. All of the jump tests are based on the principle of the stretch-shortening cycle (SSC), a crucial and inherent function that exists in human locomotion, where muscles first actively stretch before actively shortening [29,30]. The CMJ, a bipedal maximal vertical jump, was perceived in our study as the easiest and less exhausting test. This result leads to the consideration of changing the escalation protocol of our jump assessment in order that the CMJ is performed before the 10SHT instead of after it. This adjustment would ensure that fitter patients can continue with the 10 SHT and SLCMJ after the CMJ, while less fit patients perform only the CMJ. As expected, pwMS who experienced CMJ as easy, not exhausting, and safe were associated with higher CMJ performance. This suggests that positive patient experience correlates with increased lower limb neuromuscular performance during the CMJ. Furthermore, the CMJ has already been extensively analysed as a suitable test for the assessment of lower limb neuromuscular function in MS [4,14]. The previous and new findings suggest that the CMJ may therefore be very suitable for clinical routine to assess neuromuscular deficits of the lower limb in MS.

We found that higher EDSS were significantly associated with greater perceived difficulty and strain in jump performance. This is consistent with previous studies showing that patients with higher EDSS showed significantly decreased neuromuscular function of lower limb in jumping [4,12,14,15]. In contrast, no significant correlations were found between disease duration and PREM items (except self-use). This suggest that the patient experience with the jump assessment is primarily characterized by the degree of disability (EDSS) and less by the disease duration. The results also indicate that the jump assessment of the patient experience is particularly suitable for patients with a low EDSS. This is consistent with previous studies showing that the jump assessment is suitable to detect subclinical neuromuscular deficits of the lower limb below the clinical threshold of the EDSS in early MS [4,12,13].

As previous studies have shown that age, sex, and BMI have an influence on jumping performance in pwMS, it is important to investigate this when evaluating the patient experience of the jump assessment [4,14]. We found that higher age and BMI were significantly associated with greater perceived difficulty and strain in jump performance. One reason for this could be that pwMS, considering the normal aging process and an increased degenerative process, show biochemical, skeletal-muscular, and cellular changes that have a negative effect on neuromuscular function, resulting in higher perceived difficulty in jumping performance [31,32]. The results suggest that for older pwMS (50–65 years), less strenuous and difficult assessments (e.g., gait analysis) should be considered, whereas for young to middle-aged pwMS, the jump assessment is perceived as suitable. Males found the jump assessment significantly easier and less strenuous and rated the safety and comfort higher than females. One explanation for this may be that males tend to have a shorter disease course with more severe progression [33]. As a result, they may be more motivated, develop less “disease fatigue” and, because of the rapid increase in disability, may be more interested in closer monitoring for early treatment. Another explanation could be the normal growth between males and females. Males typically have a higher percentage of fast twitch (type II) fibers, which are responsible for explosive movements and high muscle strength, enabling them to jump more easily [34,35]. It is therefore clinically relevant to use the evaluation of gender-specific reference values for patient-centered monitoring when analyzing and interpreting the jump assessment in pwMS [14].

Another important result was that pwMS with fear of falling showed significantly more uncertainty and less comfort in performing the jump assessment. In addition, pwMS with falls in the previous three weeks found the SLCMJ significantly more difficult than pwMS with no falls in the previous three weeks. This could be related to the finding that pwMS with fear of falling and falls in the previous three weeks showed an increased degree of disability. Studies generally show that decreased lower limb muscle strength and balance are significant predictors of falls in pwMS [36,37]. A study in older women showed that CMJ jump height correlated with fall history [26]. Considering the lack of research on fall history in relation to jumping performance in pwMS, it was of major interest to record patients’ experience of jump assessment according to fall history and fear of falling. In addition, future studies should investigate the association between fall history, balance metrics and neuromuscular deficits in jumping performance as predictors of falls.

In addition, various sport-specific studies have shown that a high level of physical activity has a positive effect on jumping performance in adults [38,39]. Based on these influencing factors, it was also important to examine patients’ experiences in relation to physical activity, including physiotherapy. We demonstrated that pwMS receiving physiotherapy for lower limb motor deficits found the jump assessment results more useful for progress monitoring, felt safer, and rated the comfort higher than than pwMS without physiotherapy. The reason why the pwMS with physiotherapy found the CMJ and SLCMJ more difficult and strenuous is that they already have lower motor deficits because MS negatively affects skeletal muscle fiber cross-sectional area, muscle strength, and muscle mass in the lower limbs [2].

However, for the successful implementation of the sensor-based jump assessment in the standardized, patient-centered care of MS patients, it is essential to record patient-reported experiences, taking into account relevant influencing factors (e.g., clinical parameters, demographic and anthropometric characteristics, physical activity). This ensures long-term acceptance, sustainable adherence, optimization of the care process, and quality assurance [40]. In addition, the evaluation of patient-reported experiences in relation to influencing factors in pwMS is necessary for the individual interpretation of early deficits in neuromuscular function and the integration of jump assessment into diagnostic assessment in clinical practice.

PREMs are valuable tools for improving the quality of care. They support the shift towards patient-centered care and enable a more comprehensive understanding of the effectiveness and outcomes of health interventions [41]. Further research, continuous evaluation and close monitoring are needed to analyze the impact of PREMs on healthcare and clinical practice. A gradual introduction of PREMs is recommended to evaluate measurement tools and methods, ensure data quality and optimize their usability [40]. In addition, PREMs should be integrated into the evaluation of patient acceptance, satisfaction, understanding of information, confidence in treatment, and patient empowerment [42]. As patients’ experience has an impact on patients’ outcomes, long-term monitoring of PREMs should become an integral part of the healthcare service to identify and avoid problems early.

Our PREM questionnaire is a self-developed questionnaire and has been used intensively in a similar form to evaluate patient experience with the Dresden protocol for multidimensional gait analysis in a study by Scholz et al. [43,44]. For our study, the questionnaire was modified according to the jump assessment. Scholz et al. found that pwMS with low levels of disability perceived the walking assessment as less difficult and strenuous, while men and pwMS with higher levels of disability were more likely to perform it [44]. These results are consistent with ours and highlight the importance of PREMs as key indicators for the successful implementation of mobility assessments in pwMS.

Our research is not without limitations. We focused only on the patient experience, excluding the perspective of clinical staff. Including staff experience could provide a more comprehensive analysis that addresses both patient needs and the practical challenges faced by healthcare professionals, ultimately supporting more effective and sustainable implementation in clinical practice. In addition, the jump analysis was part of a visit on the day it was completed, e.g., the pwMS had a doctor’s visit, gait analysis, blood test, or similar on the same day. This may have biased the study results. Nevertheless, pwMS were asked to rate the PREM only in relation to the jump assessment. Another limitation was the small sample size of the subgroups of pwMS with fall history and fear of falling, which limited the generalizability of the findings. Future studies should include larger subgroups of participants to enable more meaningful comparisons. Although the correlations between patient experience in jump assessment and influencing factors such as age, sex, BMI, and disability were statistically significant, the correlation coefficients were generally low to moderate. Nevertheless, these results are clinically relevant and may be related to the fact that the included pwMS represent an early MS cohort (most pwMS with low EDSS 0–1.5 (42.8%), middle-age (72%) and normal weight (59.2%)). This emphasizes that, given the high acceptance of jump assessment and its potential for early detection of neuromuscular deficits, the integration of this method into routine MS monitoring—particularly in patients with mild to moderate disability—should be further explored.

## 5. Conclusions

Overall, pwMS showed a positive experience with the sensor-based jump assessment, reporting a high level of staff support performance, acceptance of required time, usefulness of the results and integration of the results in therapy. This shows that the sensor-based jump assessment is not only able to detect early motor and neuromuscular deficits in pwMS but is also a biomechanical and technology-driven suitable assessment to enable early and detailed rehabilitation approaches for physicians and patients. This is a crucial factor for the successful implementation of the sensor-based annual jump assessment, especially the CMJ, in clinical practice for continuous and patient-centered monitoring in early MS [9]. Adapting the test protocol (e.g., starting with CMJ) and addressing the specific needs of pwMS with a fear of falling or with fall history could enhance the safety, comfort, and clinical usefulness of the jump assessment.

In order to provide a more comprehensive analysis, future studies should address both the needs of patients and the practical challenges for healthcare professionals to ultimately support more effective and sustainable implementation in clinical practice.

## Figures and Tables

**Table 1:** Description of the PREM questionnaire.

	PREM Items	PREM Questions	Interpretation Scale 0–10
1	Difficulty total (and for each jump test: 10SHT, CMJ, SLCMJ)	How easy or difficult would you rate the jump assessment? (and each jump tests)	0 = easy; 10 = difficult
2	Self-use of results	To which extent do you use the results for your own review?	0 = always; 10 = not at all
3	Usefulness of the results	How useful do you think is incorporating the results into your progress monitoring?	0 = useful; 10 = not useful
4	Integration of results in MS therapy	How convinced are you that the results will be used for your disease progression?	0 = convinced; 10 = not at all convinced
5	Acceptance required time	How do you rate the time required to perform the jump assessment?	0 = appropriate; 10 = too time-consuming
6	Strain total (and for each jump test: 10SHT, CMJ, SLCMJ)	How exhausting do you experience the jump assessment?	0 = not exhausting; 10 = exhausting
7	Safety	How do you rate the safety of the jump assessment?	0 = safe; 10 = unsafe
8	Comfort	How do you rate the comfort of the jump assessment?	0 = comfortable; 10 = very uncomfortable
9	Staff support performance	How do you rate the support and explanations provided by the staff?	0 = very good; 10 = bad
10	Rating of implementation frequency	Do you think the jump assessment once a year is sufficient (if no relapses occur)?	a. jump assessment should be less frequent b. frequency is sufficient c. jump assessment should be more frequent

Abbreviations: 10SHT = 10 s hop test, CMJ = countermovement jump, SLCMJ = single-leg countermovement jump.

**Table 2:** Characteristics of the study population (N = 157). Data presented as mean (±standard deviation) unless specified otherwise.

		pwMS (N = 157)
Age (years)		39.66 (± 9.75)
Females N (%)		115 (73.2 %)
BMI		25.21 (± 5.01)
GLTEQ Score (N = 154)		41.22 (± 28.17)
Insufficiently active N (%)		25 (16.2%)
Moderate active N (%)		18 (11.7%)
Active N (%)		111 (72.1%)
Disease duration (years)		8.9 (±6.57)
MS Type N (%)	RRMS	147 (93.6%)
	PPMS	8 (5.1%)
	SPMS	2 (1.3%)
EDSS Median (IQR)	Score	2.0 (1.5–3.0)
	Pyramidal FSS	1.0 (1.0–2.0)
	Cerebellar FSS	0 (0–1.0)
	Sensory FSS	1.0 (0–2.0)
	Ambulation	0 (0–1.0)

Abbreviations: pwMS = people with Multiple Sclerosis; BMI = Body Mass Index; GLTEQ = Godin Leisure Time Exercise Questionnaire; MS = Multiple Sclerosis; RRMS = Relapsing-Remitting Multiple Sclerosis; PPMS = Primary Progressive Multiple Sclerosis; SPMS = Secondary Progressive Multiple Sclerosis; EDSS = Expanded Disability Status Scale; FSS = Functional System Score; IQR = Interquartile Range.

**Table 3:** Correlation between PREM items and continues variables sex, age, BMI, physical activity, and clinical outcomes in pwMS (n = 157) according to Spearman.

PREM Items	Sex	Age	BMI	Physical Activity	Disease Duration	EDSS Score	FSS Pyramidal	FSS Cerebellar	FSS Sensory	Ambulation Score
Difficulty total	0.167 *	0.093 *	0.110 *	0.093 *	−0.077	0.099 *	0.207 *	0.167 *	0.095	0.185 *
10SHT	0.071	0.213 *	0.085	−0.057	0.028	0.235 *	0.312 *	0.246 *	0.072	0.302 *
CMJ	0.187 *	0.242 *	0.064	−0.081	−0.002	0.228 *	0.283 *	0.271 *	0.156 *	0.257 *
SLCMJ	0.168 *	0.156 *	0.200 *	−0.143 *	0.031	0.136 *	0.226 *	0.120 *	0.077	0.126 *
Self-use of results	0.103 *	−0.03	0.103 *	−0.013	0.141 *	−0.024	−0.035	−0.086	0.014	0.060
Usefulness of the results	0.141 *	−0.144 *	0.059	−0.060	0.066	−0.062	−0.009	−0.165 *	−0.034	0.052
Integration of results in therapy	0.250 *	−0.081	0.000	0.088	0.055	−0.034	0.04	−0.056	−0.007	−0.035
Acceptance required time	0.080	0.013	−0.010	0.084	−0.010	0.043	0.124 *	0.042	0.031	0.027
Strain total	0.215 *	0.118 *	0.237 *	−0.093	0.048	0.200 *	0.247 *	0.170 *	0.068	0.200 *
10SHT	0.152 *	0.190 *	0.081	−0.155 *	−0.038	0.266 *	0.299 *	0.217 *	0.062	0.283 *
CMJ	0.258 *	0.255 *	0.162 *	−0.123 *	0.004	0.291 *	0.362 *	0.334 *	0.194 *	0.386 *
SLCMJ	0.258 *	0.186 *	0.197 *	−0.121 *	0.065	0.252 *	0.323 *	0.218 *	0.087	0.292 *
Safety jump assessment	0.282 *	0.100 *	0.169 *	−0.187 *	0.036	0.272 *	0.326 *	0.250 *	0.130 *	0.340 *
Comfort jump assessment	0.209 *	−0.067	0.095 *	−0.161 *	0.031	0.023	−0.026	−0.073	0.031	0.041
Staff support performance	0.117 *	0.017	−0.035	−0.233 *	0.083	0.091	0.139 *	0.045	0.029	0.114 *
Rating of implementation frequency	0.220 *	0.041	−0.054	0.129 *	−0.016	0.104 *	0.07	0.063	0.103 *	0.110 *

* = significant correlation (*p* < 0.05). Abbreviations: 10SHT = 10 s hop-test, CMJ = countermovement jump, SLCMJ = single-leg countermovement jump, BMI = body mass index, EDSS = Expanded Disability Status Scale. FSS = functional system score.

**Table 4:** Association between PREM items and CMJ parameters in pwMS (n = 157) according to Spearman correlation.

	Peak Force (N/kg)	Peak Power (W/kg)	Force at Zero Velocity (N/kg)	Flight Time (s)	Flight Time-Contraction Time Ratio	Jump Height (cm)	Propulsive Time (s)	Braking Time (s)	Negative Work (J)	Positive Work (J)
Difficulty CMJ	−0.308 *	−0.461 *	−0.148 *	−0.430 *	−0.287 *	−0.448 *	0.053	0.029	0.064	−0.264 *
Self-use of results	−0.216 *	−0.247 *	−0.085	−0.224 *	−0.194 *	−0.222 *	0.106 *	0.018	0.005	−0.007
Usefulness of the results	−0.104 *	−0.187 *	−0.033	−0.207 *	−0.128 *	−0.190 *	0.031	−0.077	0.093 *	−0.054
Integration of results in therapy	−0.123 *	−0.216 *	−0.026	−0.202 *	−0.121 *	−0.231 *	0.005	−0.019	0.063	−0.139 *
Acceptance required time	0.046	−0.076	0.067	−0.072	0.031	−0.099 *	−0.113 *	−0.119 *	0.074	−0.113 *
Strain CMJ	−0.353 *	−0.472 *	−0.201 *	−0.465 *	−0.319 *	−0.465 *	0.087	0.012	0.089	−0.203 *
Safety	−0.329 *	−0.411 *	−0.0232 *	−0.433 *	−0.359 *	−0.417 *	0.147 *	0.050	0.087	−0.160 *
Comfort	−0.105 *	−0.221 *	−0.049	−0.210 *	−0.137 *	−0.232 *	−0.009	−0.021	0.054	−0.127 *
Staff support performance	0.069	−0.112 *	0.098 *	−0.139 *	−0.020	−0.133 *	−0.143 *	−0.165 *	0.134 *	−0.176 *
Implementation frequency	0.045	0.139 *	−0.037	0.132 *	0.016	0.125 *	0.014	0.054	−0.018	0.014

* = significant correlations, Abbreviation: CMJ = countermovement jump.

## Data Availability

All data produced in the present study are available upon reasonable request to the authors.

## Appendix A

**Table A1:** Detailed description of the PREM Items regarding to physiotherapy, fear of falling, and falls in the previous three weeks (median (IQR)).

	Physiotherapy		Fear of Falling		Falls in the Previous Three Weeks	
PREM Items	PwMS with Physiotherapy (N = 42)	PwMS without Physiotherapy (N = 106)	PwMS with Fear of Falling (N = 22)	PwMS without Fear of Falling (N = 126)	PwMS with Falls in the Previous 3 Weeks (N = 9)	PwMS Without Falls in the Previous 3 Weeks (N = 139)
Performance difficulty total	3 (2–5) ^A^	1 (0–2) ^A^	2 (1–6) ^B^	1 (0–3) ^B^	2 (1–4)	2 (0–3)
Self-use of results	2 (1–5)	2 (0–5)	3 (1–5)	2 (0–5)	2 (1–3) ^C^	5 (4–6) ^C^
Usefulness of the results	1 (0–1) ^A^	0 (0–1) ^A^	0 (0–1)	0 (0–2)	1 (0–1)	0 (0–2)
Integration of results in therapy	1 (0–1) ^A^	0 (0–1) ^A^	0.5 (0–2)	0 (0–1)	0 (0–1)	0 (0–2)
Acceptance required time	0 (0–0)	0 (0–1)	0 (0–1)	0 (0–0)	0 (0–0)	0 (0–0.75)
Strain total	4 (2–6) ^A^	2 (1–3) ^A^	5 (4–6) ^B^	2 (1–3) ^B^	3 (0–4)	3 (1–4)
Safety	3 (2–5) ^A^	1 (0–3) ^A^	5 (4–6) ^B^	1 (0–3) ^B^	3 (1–5)	2 (0–3)
Comfort	1 (0–2) ^A^	1 (0–2) ^A^	2 (1–3) ^B^	1 (0–2) ^B^	0 (0–1)	1 (0–2)
Staff support performance	0 (0–0)	0 (0–0)	1 (1–2) ^B^	0 (0–0) ^B^	0 (0–0)	0 (0–0)
Rating of implementation frequency *	5 (5–10)	5 (5–5)	5 (5–5)	5 (5–5)	7.5 (5–10) ^C^	5 (5–5) ^C^

^A^ = significant difference between pwMS with and without physiotherapy (*p* < 0.05). ^B^ = significant difference between pwMS with and without fear of falling (*p* < 0.05). ^C^ = significant difference between pwMS with and without falls in the previous 3 weeks (*p* < 0.05). * = Do you think the jump assessment once a year is sufficient? (0 = less frequent, 5 = sufficient, 10 = more frequent).

**Table A2:** Detailed description of the PREM Items regarding to sex, age, EDSS, physical activity, and BMI subgroups (median (IQR)).

PREM	Sex		Age			EDSS			Physical Activity			BMI		
	Female (N = 115)	Male (N = 42)	Young (18–30 Years) (N = 15)	Middle-Age (31–49 Years) (N = 113)	Old (50–65 Years) (N = 29)	EDSS 0–1.5 (N = 67)	EDSS 2–3 (N = 60)	EDSS 3.5–5.5 (N = 30)	Active (N = 111)	Moderate (N = 18)	Insufficient (N = 25)	Normal Weight (18–25) (N = 93)	Overweight (25–30) (N = 40)	Obesity (30+) (N = 24)
Performance difficulty total	2 (1–3)	1 (1–2)	2 (1–2)	1 (0–2)	2 (1–5)	2 (1–3)	1 (1–3)	2 (1–5)	1 (0–3)	2 (1–3)	2 (0–3)	1 (0–3)	2 (1–3)	2 (0–3)
10SHT	2 (1–3)	2 (0–3)	2 (1–2)	2 (1–3)	3 (1–5)	1 (0.75–2)	2 (0–3)	3 (1–6)	2 (0–4)	2 (1–5)	2 (1–3)	2 (0–3)	2 (1–4)	1 (0–3)
CMJ	2 (1–3)	1 (0–2)	2 (1–2)	2 (1–3)	2 (0–5)	1 (0–2)	1 (0–3)	3 (1–5)	2 (0–3)	1 (1–2)	1 (0–3)	1 (0–3)	2 (0.5–3)	1 (0–3)
SLCMJ	4 (2–6)	3 (1–4.5)	4 (2–6)	2 (1–5)	4 (1–8)	3 (1–5)	3 (2–5)	5 (1.5–8)	5 (3–6)	3 (1.5–5.5)	3 (2–5)	3 (1.5–5)	3 (2–6)	5 (2–7)
Self-use of results	2 (0–5)	2 (0–4)	3 (1–3)	2 (1–3)	2 (1–3)	2 (0.75–5)	2 (0–4)	4 (0–2)	2 (0–4.5)	2.5 (0–5)	2 (0–5)	2 (0–4)	2 (0–5)	3 (1–7)
Usefulness of the results	0 (0–2)	0 (0–1)	1 (0–4)	0 (0–1)	3 (1–5)	0 (0–2)	0 (0–1)	0 (0–1)	0 (0–2)	0.5 (0–2)	0 (0–1)	0 (0–1)	0 (0–2)	0 (0–1.5)
Integration of results in therapy	0 (0–2)	0 (0–1)	1 (0–4)	0 (0–2)	1 (1–3)	0 (0–2)	0 (0–1)	0 (0–2)	0 (0–1)	0 (0–2)	0 (0–2)	0 (0–1)	0 (0–1)	0 (0–2)
Acceptance required time	0 (0–1)	0 (0–0)	3 (1–4)	2 (1–3)	3 (1–5)	0 (0–0)	0 (0–1)	0 (0–1)	0 (0–0)	0 (0–1)	0 (0–1)	0 (0–1)	0 (0–0.5)	0 (0–0)
Strain total	3 (1–5)	2 (0–3)	2 (1–3)	2 (1–2)	3 (1–5)	2 (1–3)	2 (1–4)	3 (2–6)	3 (1–5)	2 (1–5)	3 (1–4)	2 (1–3)	3 (1–4)	3 (2–5)
10SHT	2 (1–4)	1 (0–3)	1 (0–2)	1 (0–2)	4 (3–5.75)	1 (1–3)	2 (1–4)	4 (2–6)	3 (1–4)	2.5 (1–6)	2 (1–4)	2 (1–4)	2 (1–5)	2 (1–5)
CMJ	2 (1–4)	1 (0–2)	2 (1–2)	2 (1–3)	5 (3–6.75)	2 (1–3)	2 (1–3)	4 (2–6)	2 (1–3)	2 (1–3)	2 (1–3)	2 (1–3)	2 (1–3)	3 (1–5)
SLCMJ	4 (2–6)	3 (1–4)	3 (2–5)	3 (2–4)	5 (3–7)	3 (1–4)	4 (2–6)	6 (3–8)	5 (3–6)	3 (2–4)	3 (2–5)	3 (2–5)	3 (2–5)	4 (2–6)
Safety jump assessment	2 (1–4)	1 (0–2)	2 (1–3)	1 (0.25–3)	3 (0.5–5)	1 (0–3)	1 (0–3)	4 (2–6)	3 (1–4)	2 (1–4)	1 (0–3)	1 (0–3)	2 (1–3)	2 (1–5)
Comfort jump assessment	1 (0–2)	0 (0–1)	1 (0–1)	1 (0.25–3)	0.5 (0–2)	1 (0–2)	1 (0–2)	1 (0–2)	1 (0–2)	1 (0–2)	0 (0–2)	0 (0–2)	1 (0–2)	1 (0–2)
Staff support performance	0 (0–0)	0 (0–0)	0 (0–0)	2 (0–3)	0 (0–3)	0 (0–0)	0 (0–0)	0 (0–0)	0 (0–0)	0 (0–0)	0 (0–0)	0 (0–0)	0 (0–0)	0 (0–0)
Rating of implementation frequency *	5 (5–5)	5 (5–5)	5 (0–5)	5 (0–5)	5 (0–5)	5 (5–5)	5 (5–5)	5 (5–5)	5 (5–5)	5 (5–5)	5 (5–5)	5 (5–5)	5 (5–5)	5 (5–5)

Abbreviations: 10SHT = 10 s hop-test, CMJ = countermovement jump, SLCMJ = single-leg countermovement jump, BMI = body mass index, EDSS = Expanded Disability Status Scale. * = Do you think the jump assessment once a year is sufficient? (0 = less frequent, 5 = sufficient, 10 = more frequent).
